# The role of inner nuclear membrane proteins in tumourigenesis and as potential targets for cancer therapy

**DOI:** 10.1007/s10555-022-10065-z

**Published:** 2022-10-07

**Authors:** Maddison Rose, Joshua T. Burgess, Kenneth O’Byrne, Derek J. Richard, Emma Bolderson

**Affiliations:** 1 Cancer & Ageing Research Program (CARP), Centre for Genomics and Personalised Health (CGPH), School of Biomedical Sciences, Queensland University of Technology (QUT), Brisbane, QLD Australia; 2 Princess Alexandra Hospital, Ipswich Road, Woolloongabba, Brisbane, QLD 4102 Australia

**Keywords:** Cancer, Nuclear envelope, Cancer therapeutics, Lamin, Lem-Domain protein, Banf1

## Abstract

Despite significant advances in our understanding of tumourigenesis and cancer therapeutics, cancer continues to account for 30% of worldwide deaths. Therefore, there remains an unmet need for the development of cancer therapies to improve patient quality of life and survival outcomes. The inner nuclear membrane has an essential role in cell division, cell signalling, transcription, cell cycle progression, chromosome tethering, cell migration and mitosis. Furthermore, expression of several inner nuclear membrane proteins has been shown to be frequently altered in tumour cells, resulting in the dysregulation of cellular pathways to promote tumourigenesis. However, to date, minimal research has been conducted to investigate how targeting these dysregulated and variably expressed proteins may provide a novel avenue for cancer therapies. In this review, we present an overview of the involvement of the inner nuclear membrane proteins within the hallmarks of cancer and how they may be exploited as potent anti-cancer therapeutics.

## Introduction

Cancer defines a broad category of diseases, defined by rapid, uncontrolled and dysregulated cellular growth. On average, there are 442 new cases of cancer diagnosed each year per 100,000 people and cancer related deaths account for ~ 30% of worldwide deaths [1]. Conventional anti-cancer therapies consist largely of chemotherapy, radiation and surgical approaches. However, these approaches are often associated with high rates of tumour reoccurrence and poor patient quality of life outcomes during treatment [2]. For instance, chemotherapeutic agents target rapidly dividing cells, rather than specifically targeting those of a cancerous origin. This includes cells of the intestinal epithelium, bone marrow and epidermal cells. Consequently, they induce side effects such as hair loss, nausea and myelosuppression [3]. There has since been significant improvements in cancer therapeutics, with a large focus on personalised and targeted anti-cancer therapeutics, including hormonal and immunotherapy approaches (Reviewed in [4]). However, tumours rapidly mutate to develop mechanisms which are able to limit the effectiveness of these treatments. Therefore, the exploration of novel therapeutic options is essential to increase the survival and quality of life of cancer patients.

In early literature, the nuclear envelope was simply defined as a physical barrier and later was suggested to protect the nuclear genomic DNA from damage and degradation by the cytoplasmic cellular components [5]. However, it is now widely recognised that the nuclear envelope, and its associated proteins, have imperative roles in an array of cellular processes. These include cell division, cell signalling, transcription, cell cycle progression, chromosome tethering, cytoplasm-nuclear transport and cell migration. Furthermore, organised breakdown and reformation of the nuclear envelope is essential for mitosis to occur. Nuclear envelope breakdown, mitosis and the subsequent reformation of the nuclear envelope are complex processes, involving the cooperation of many nuclear envelope proteins. For nuclear envelope breakdown to occur, several nuclear envelope proteins, including numerous Nucleoporins, Lamin A/B, Lap2 and Banf1, must be hyperphosphorylated by kinases to disrupt protein:protein interactions and promote NPC disassembly, lamina depolymerisation and the dissociation of chromatin from the nuclear envelope [6–11]. Several nuclear envelope proteins have also been shown to be directly involved in mitosis itself. For instance, siRNA-mediated depletion of Lamin B has been shown to induce mitotic spindle defects [12]. Similarly, for nuclear envelope reformation to occur, dephosphorylation of several nuclear envelope proteins is required, including Banf1, Lem4 and Lamin A [13]. Dephosphorylation of Nups is also required for reconstruction of nuclear pore complexes in the newly reformed nuclear envelope. In addition, Lem2 is required to recruit CHMP7, subsequently triggering ESCRT-III polymerisation to promote microtubule-severing and nuclear envelope reformation [14].

**Fig. 1:**
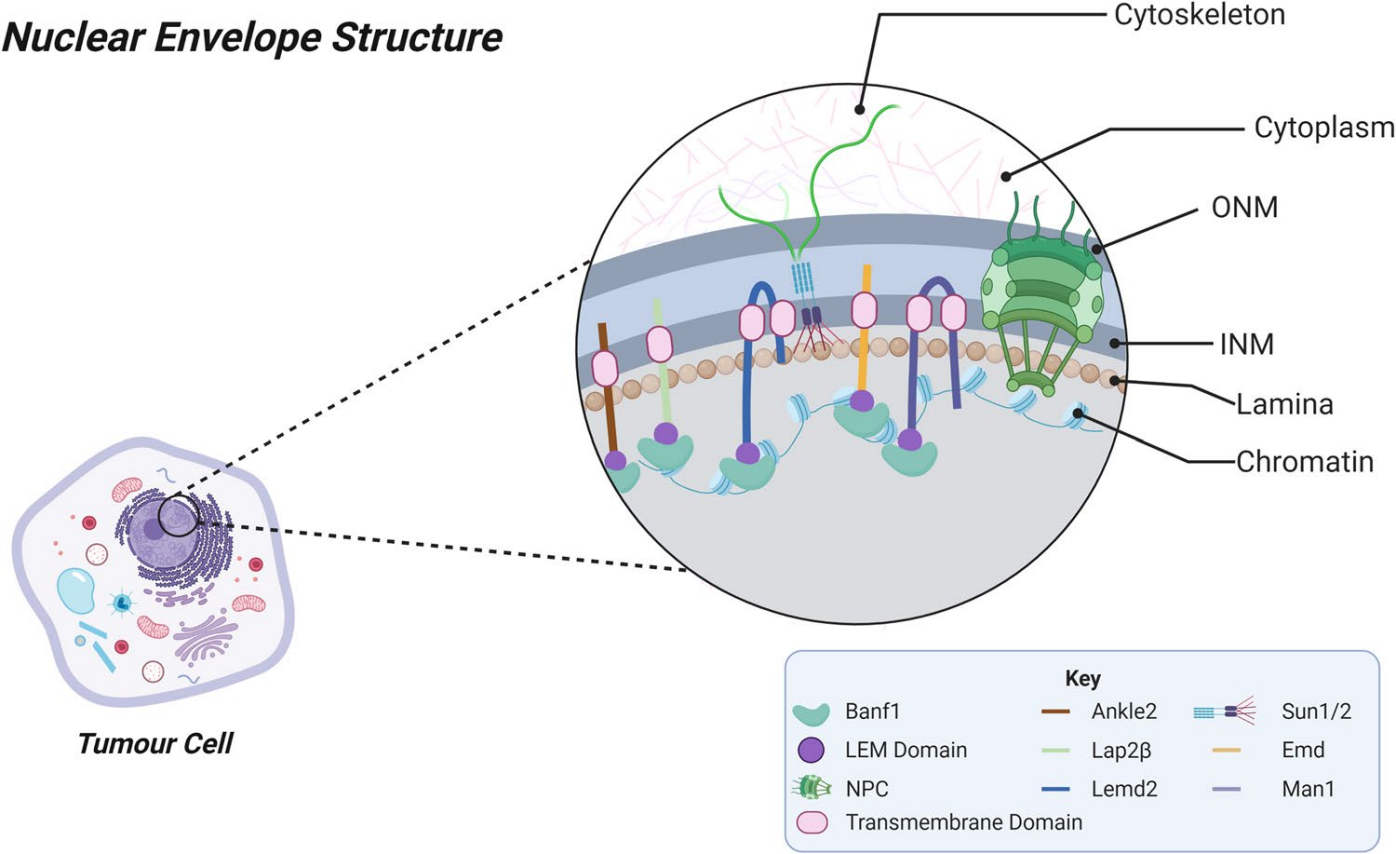
Nuclear envelope structure

At its most fundamental level, the nuclear envelope is a double lipid membrane which encapsulates the nucleus to generate structural integrity within the nucleus (Fig. 1). The nuclear envelope can be further subcategorised as the inner nuclear membrane (INM) and outer nuclear membrane (ONM). The ONM is considered as structurally and functionally similar to the membrane of the endoplasmic reticulum (ER), and the proteins of the ONM include KASH proteins and Nesprins. In addition to acting as a physical barrier between the nucleus and the cytoplasm, the ONM and its proteins are also involved in cell signalling, forming connections with the cytoskeleton and maintaining appropriate nuclear localisation within the cell [15]. Whereas the INM is described as being both structurally and functionally distinct to the ONM and ER membrane. The INM is also made up of a unique group of proteins, including lamins, Lem domain proteins, Banf1, SUN domain proteins and the Lamin B receptor. The INM and its proteins are involved in mitosis, gene transcription, the cell cycle and several other cellular processes [16, 17].

These membranes are separated by a ~ 50 nm perinuclear space, which is continuous with the internal component of the endoplasmic reticulum. Underlying the inner nuclear membrane is the nuclear lamina, consisting of a fibrous network of Lamins which is largely responsible for maintaining nuclear rigidity (Reviewed in [18]). Selective, bidirectional exchange between the nucleus and cytoplasm is enabled by passive diffusion or energy-dependent transport via nuclear pore complexes (NPCs). More specifically, the nuclear pore complexes are relatively large subcellular structures consisting of 50–100 unique nucleoporins (Nups). NPCs allow for nucleocytoplasmic transport of small polar molecules, ions, proteins and RNAs (reviewed in [19]). The NPC are also known to be widely involved in cellular functioning. It has been suggested that NPCs are involved in gene transcription, by associating with active genes to facilitate mRNA export out of the nucleus [20]. Several works have supported this theory, demonstrating that NPCs physically associate with activated genes, regulating their spatial organisation and expression. However, the exact mechanism by which NPCs are involved in transcriptional regulation remains to be fully established [21]. The NCP have also been suggested to be involved in DNA repair, with a specific subtype of DNA lesions relocalising to NPCs [22].

The nuclear envelope, and its encompassing proteins, have been shown to be required for an extensive list of cellular functions. Therefore, it is not surprising that dysregulation or mutation of numerous nuclear envelope proteins has been shown to have a role in the progression of various diseases, including premature ageing syndromes, cardiac conditions, neurological conditions, muscular dystrophy and cancer [23–26]. For instance, mutations in the LMNA gene which encodes Lamin A/C have been shown to induce several diseases with a diverse range of clinical presentations [27]. A mutation in the LMNA gene has been shown to induce familial partial lipodystrophy of the Dunnigan type (FPLD). FPLD clinically presents as lipoatrophy of the limbs and trunk, fat accumulation in the neck and face and is often associated with insulin-resistant diabetes [28]. LMNA gene mutations have also been linked to Limb-Girdle muscular dystrophy (LGMD1B), which presents as progressive weakening and wasting of the limb muscles, joint contractures and several cardiac disturbances [29]. Mutation of the LMNA gene has also been shown to induce the neurological condition, Charcot Marie tooth disease (Type 2B1), which induces lower motor neuron lesions that inevitably produce abnormal gate patterns and pretibial and peroneal muscle weakness [30]. Mutation of Lemd3 has been shown to be associated with Buschke-Ollendorff syndrome, characterised by osteopoikilosis and connective tissue nevi [31]. Furthermore, mutations in several nuclear envelope genes have been shown to be associated with progeria syndromes, including Banf1, Lamin A/C and Lemd2 [23, 25, 32, 33].

## The nuclear envelope in cancer

Abnormal nuclear morphology is a well-recognised characteristic amongst tumour cells. In fact, the Papanicolaou’s smear test (Pap smear) was developed in 1949 as a diagnostic technique for cervical cancer, following the observation that cervical carcinoma cells frequently possess a non-spherical nucleus [34]. Aberrations in the structure and expression of nuclear envelope proteins have also been strongly linked to abnormal nuclear envelope morphology [35]. Therefore, it is unsurprising that aberrant expression or activity of nuclear envelope proteins has been linked to disruption of other cellular processes. It has been suggested that targeting various inner nuclear membrane proteins may impair tumour cell growth and migration by a number of mechanisms, including inhibiting tumour cell growth and metastasis by disruption of NE reformation after mitosis; and promoting NE ruptures and preventing their repair, leading to tumour cell death [36]. Targeting NE proteins may also increase genomic instability via exposure of DNA to the cytoplasm and inhibition of DNA repair, leading to irreparable DNA damage and subsequent tumour cell death.

**Fig. 2:**
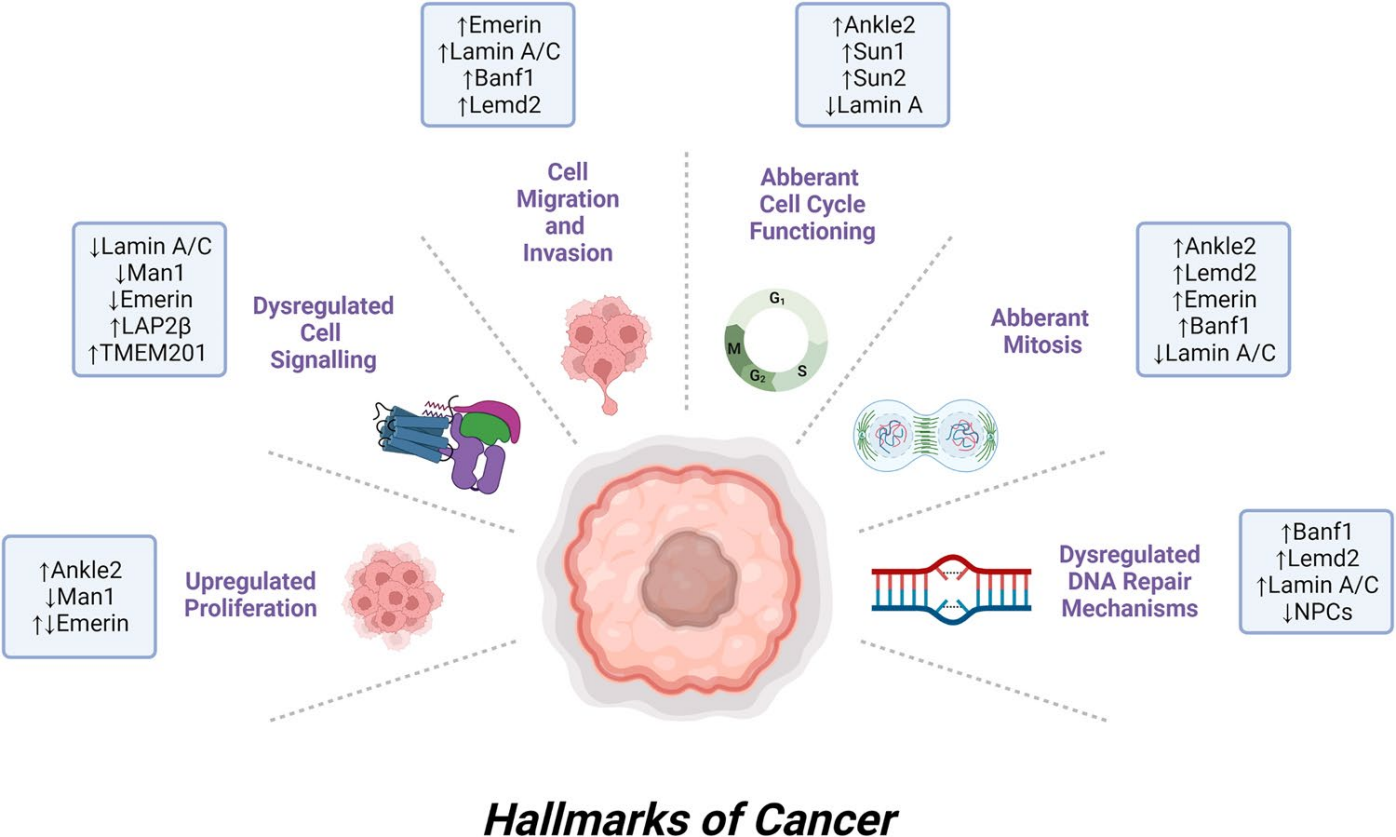
Hallmarks of cancer

The hallmarks of cancer are a well-recognised group of biological capabilities acquired by cancer cells to aid their unchecked and persistent proliferative capacity [37]. This review discusses the role of the nuclear envelope proteins in the promotion of these hallmarks, and whether these proteins may be targeted as an anti-cancer therapy (Fig. 2).

## The involvement of nuclear envelope proteins in tumour growth and metastasis

### Cell migration and invasion

In addition to their roles in establishing and maintaining normal cellular structure, the nuclear envelope and associated proteins have been shown to have roles in tumour cell migration and invasion. Cellular migration and invasion are two essential factors in the metastatic cascade, where tumours have the capacity to spread from their primary site [38]. Understanding the proteins which have a key role in these pathways will not only improve our knowledge of tumour metastatic pathways, but also allow for the exploration of anti-cancer therapies which minimise the capacity for tumours to metastasise.

Recent research has demonstrated that alterations in nuclear conformation and subcellular localisation are vital for effective migration to occur. More specifically, nuclear movement secondary to protrusion of the cell’s edge by actin filaments is essential for cellular migration [38]. Several nuclear envelope proteins have been shown to have a vital role in maintaining the appropriate cytoskeletal actin structure and nuclear movement to promote cellular migration [39, 40]. It has been demonstrated that Emerin-mediated nuclear/myosin IIB coupling is essential to achieve directional flow of actin filaments during cellular migration [39]. Similarly, A-type Lamins have been shown to anchor nesprin-2G-SUN2 TAN lines to the nucleus to ensure appropriate nuclear positioning for migratory pathways [40]. Furthermore, depletion of these proteins by siRNA has been shown to impair this subcellular movement to impair cellular migration [39, 40]. Thereby, inhibition of these proteins may provide a favourable anti-cancer therapy by diminishing the capacity of tumour cells to enter the appropriate morphology for proficient migration, inevitably reducing the metastatic capacity of these tumours.

Movement of the nucleus is often considered the rate-limiting step during cellular migration. This is predominately due to the observation that cytoplasmic components of the cell can squeeze through minute spaces; however, the nucleus has significantly more limited flexibility [41]. Therefore, it is unsurprising that there are a substantial number of interphase nuclear rupture events during cellular migration due to the extensive mechanical force placed on the nucleus [42, 43]. Furthermore, Banf1 and the A-type Lamins have been shown to have an essential role in maintaining cellular integrity during migration with pressure required to induce nuclear ruptures being significantly less following depletion of Banf1 or the A-type Lamins [44].

Recently, substantial research has been conducted to further understand the mechanism by which cells repair these mechanical-stress induced ruptures. The key nuclear envelope protein, Banf1, has been shown to be essential for the rapid repair of these ruptures [42]. Immediately following rupture, cytosolic Banf1 rapidly relocalises to the rupture site due to Banf1’s affinity for double-stranded DNA [45]. Subsequently, this promotes the recruitment of other nuclear envelope repair proteins, including ESCRT III and the Lem domain protein, Lemd2, to the site of rupture [44]. Hence, inhibition or loss of Banf1 impairs nuclear envelope rupture repair; therefore, inducing catastrophic damage and subsequent cell death in migrating cells. Secondary to this, Banf1’s ability to bind dsDNA outcompetes that of cyclic guanosine monophosphate–adenosine monophosphate synthase (cGAS). cGAS is a double-stranded DNA (dsDNA) sensor, usually localised to the cytoplasm, essential for activation of stimulator of interferon genes (STING). Therefore, if cells were unable to protect their dsDNA from cGAS in the event of a nuclear rupture, this would likely result in a catastrophic event and subsequent cell death [46, 47].

Lamin A/C has an essential role in upholding nuclear stability whilst maintaining some level of flexibility to minimise catastrophe following the mechanical stress of cellular migration [48]. Supporting this, one study demonstrated that depletion of Lamin A/C decreased prostate cancer cell colony formation, migration and invasion due to upregulation of the PI3K/AKT cell survival pathway [49]. Inversely, over-expression of Lamin A was shown to promote invasion and migration in a colorectal cancer model due to the upregulation of T-plastin and subsequent downregulation of E-cadherin [50]. In contrast, another study found that expression of Lamin A/C has been shown to be negatively correlated with the probability of developing distant metastatic lesions in breast cancer. Specifically, depletion of Lamin A was shown to enhance confined cell migration capacity in breast cancer models by creating highly deformable nuclei [24]. These opposing findings suggest that Lamin A/C expression may have a multifaceted role in tumour progression, which is likely mediated by external factors.

Collectively, these findings suggest that targeting various nuclear envelope proteins may impair tumour cell migration, thereby decreasing the risk of metastatic sites by a magnitude of mechanisms. The targeting of nuclear envelope proteins may have several multifaceted effects on tumour cell migration including inhibiting subcellular re-localisation of the nucleus to prevent the initiation of migration, preventing the repair of migration induced nuclear envelope ruptures and disrupting the precise regulation of nuclear stability to promote nuclear envelope ruptures which subsequently cannot be repaired leading to tumour cell death.

### Cell cycle and division

Aberrant cell cycle activity is characteristic amongst tumours; therefore, cell cycle inhibitors have shown promise in the development of anti-cancer therapeutics. However, the current clinically available cell cycle kinase inhibitors have substantial drawbacks. These include unsustainable neurological and bone marrow off target toxicity, a high prevalence of acquired resistance and poor improvements in patient outcomes (reviewed in [51]). The nuclear envelope is well recognised to have an essential role in allowing for changes in nuclear shape and size throughout the cell cycle. Therefore, it is possible that down regulating the expression or activity of key nuclear envelope proteins, may act to inhibit cell cycle progression and subsequently to suppress tumour cell growth. Furthermore, targeting these nuclear envelope proteins may have less off-target effects than conventional cell cycle therapies given their altered expression in tumours.

Recently, a novel role was revealed for the Lem domain protein, Ankle2, in breast cancer tumourigenesis. They demonstrated that Ankle2 is overexpressed in breast cancer, and this overexpression facilitated breast cancer proliferation and migration. This effect on proliferation was suggested to be through promotion of the transition through the G1 to S phase of the cell cycle via the stabilisation of Erα, induction of Erα transactivation activity and promotion of Aurora A-mediated phosphorylation of Erα [17]. Based on these observations, it can be hypothesised that compounds which supress Ankle2 levels or activity may provide favourable anti-tumour activity like that of a traditional cell cycle checkpoint inhibitor. Although, given Ankle2 is substantially overexpressed in tumour cells, it is possible that specifically targeting Ankle2 may have substantially less off-target toxicity than conventional therapies.

In addition, in mouse embryotic fibroblast models, Sun1 and Sun2 null mice demonstrated significant anti-proliferative effects due to the accumulation of cells within the S phase of the cell cycle [52]. Therefore, this suggests that targeting the expression or activity of these proteins may have positive implications for tumour patients. However, the implications of this for a tumorigenic model remain to be investigated.

Similarly, several nuclear envelope proteins have been shown to have a role in the mitotic phase of the cell cycle [36, 53–55]. This is largely because nuclear envelope breakdown and reformation is essential for mitosis to occur [56]. The exploitation of compounds which disrupt mitosis as a cancer therapy has been of scientific interest for many years, with the intent of directly inhibiting the production of new tumour cells. However, most current anti-mitotic therapeutics, such as paclitaxel and docetaxel, target microtubule polymerisation dynamics and induce substantial off-target toxicity due to their inability to distinguish between tumour cells and other highly proliferative non-tumorigenic cell types [57]. Although there is yet to be a cancer therapeutic developed, which targets the involvement of nuclear envelope proteins in mitosis, recent research has demonstrated that this may be a viable target. For instance, it has also been shown that the phosphorylation state of Banf1 is key in the regulation of cell division [36]. During mitotic entry, VRK1 phosphorylates Banf1 resulting in Banf1 being unable to maintain binding with Lem domain proteins and DNA. Thereby, this promotes the dissociation of chromosomes from the nuclear envelope during prophase. Likewise, at the termination of cell division, the nuclear envelope localised Lem domain protein, Lem4, binds to VRK1 to promote the dephosphorylation of Banf1 by PP2A allowing for the reformation of the nuclear envelope [9, 16]. Thereby, either directly or indirectly suppressing Banf1’s role in nuclear envelope breakdown and reformation may provide a novel anti-mitotic drug without inducing the off-target effects associated with the currently available therapeutics.

Similarly, in HeLa cervical cancer cells, the Lem domain protein, Emerin, was shown to relocalise to the centrosomes and microtubules during mitosis [58]. In addition, deletion of various regions of the gene encoding Emerin induced several mitosis deficient phenotypes, including tubulin network and centromere mislocalisation and prolonged time in mitosis, suggesting that Emerin is required for appropriate mitotic functioning [59]. Thereby, targeting Emerin’s role in mitosis as an anti-cancer therapy may have similar activity to that of targeting Banf1.

As previously discussed, Lamin A/C expression is frequently downregulated in tumour cells [50, 60]. In addition to having a role in tumour cell migration and invasion, the downregulation of Lamin A/C has also been shown to induce aberrations in mitosis [48, 60]. More specifically, siRNA-mediated depletion of Lamin A/C was shown to induce nuclear budding, tripolar cell division, aneuploid cells and micronuclei in ovarian carcinoma cells [60]. Taken together, these studies also implicate targeting Lamin A/C as a potential anti-cancer strategy.

### Cell signalling

Dysregulation of cell signalling cascades is well recognised to be a shared characteristic amongst cancers. It is well recognised that several nuclear envelope proteins are involved in cell signalling pathways. For example, the MAPK/ERK pathway is a complex cellular pathway with extensive involvement in cellular proliferation, apoptosis, and differentiation [61]. Dysregulated and hyper-activation of MAPK/ERK signalling has shown to promote tumourigenesis via several mechanisms (reviewed in [62]). The role of several nuclear envelope proteins in maintaining appropriate MAPK/ERK signalling has been established. For instance, it has been shown that siRNA-mediated depletion of Lamin A/C or Emerin in cervical cancer cells results in increased ERK phosphorylation and localisation to the nucleus, subsequently enabling activation of downstream transcription factors [63, 64].

Another instance of nuclear envelope proteins being involved in cell signalling is the role of the Lem domain protein, MAN1, in the transforming growth factor-β (TGF-β) signalling pathway [65]. The TGF-β pathway has significant involvement in many cellular pathways including proliferation, differentiation, adhesion and cell cycle regulation. Like the MAPK/ERK pathway, TGF-β is frequently dysregulated in cancer cells [66]. MAN1 has been shown to bind two intracellular mediators of TGF-β signalling, Smad2 and Smad3, to suppress their ability to stimulate TGF-β activity. Subsequent studies showed that MAN1 overexpression inhibits the nuclear translation of Smad2 and Smad3, consequently, inhibiting cellular proliferation [65, 67]. Despite these findings, it remains to be determined if MAN1 can be targeted as an anti-cancer therapy. The transmembrane inner nuclear membrane protein, TMEM201, has also been shown to have a role in regulation of TGF-β and Smad2/3, suggesting that multiple inner nuclear membrane proteins may regulate this pathway [68].

Upregulation of the Wnt signalling pathway has been well established to be a shared characteristic amongst many tumour types, resulting in favourable conditions for the unregulated proliferation of tumour cells [69, 70]. Emerin, an inner nuclear membrane protein, has been shown to downregulate the activity β-catenin by inhibiting its nuclear accumulation via the upregulation of β-catenin export to the cytoplasm. The accumulation of β-catenin in the nucleus inevitably enables the activation of transcription factors, thereby promoting Wnt signalling [71]. Emerin has been shown to be dynamically expressed in a wide variety of tumours, with its expression being significantly decreased in highly metastatic tumours [72]. Therefore, it is possible that the downregulation of Emerin increases Wnt signalling by minimising nuclear export of β-catenin, thereby promoting cellular migration and invasion [72].

In addition, LAP2β has been shown to be overexpressed in a variety of tumours including gastric, stomach, breast, and lung cancers [73, 74]. Like other nuclear envelope proteins, LAP2β has been shown to participate in several cell signalling pathways, including suppression of the transcriptional activity of various transcription factors. The role of p53 as a transcription factor in tumourigenesis is well documented, with p53 having an essential role in the cellular stress response to promote DNA repair, cell cycle arrest, cellular senescence and apoptotic mechanisms (reviewed in [75]). Of interest, LAP2β expression has been shown to inversely correlate with p53 transcriptional activity [76]. Thereby, the characteristic overexpression of LAP2β in tumour cells is suggested to have a role in supressing the anti-tumour activity of p53. However, it is arguable that that this may not be of therapeutic significance as greater than 50% of solid tumours have p53 mutations.

### DNA damage response and genome stability

Finally, DNA repair defects are characteristic amongst a large subset of tumours. Several nuclear envelope proteins have been shown to participate in the cellular response to DNA damage, including Lamin A/C, Lemd2 and Banf1 [77–79].

As previously discussed, Lamin A/C expression is frequently altered in tumour cells. Furthermore, Lamin A/C has been shown to be involved in numerous DNA damage response pathways, with its central role in DSB repair being the most studied. Briefly, HR is an error-free and template driven DNA double strand break repair method, that is limited to the S and G2 phases of the cell cycle. Whereas NHEJ can occur throughout all phases of the cell cycle as it does not rely on a sister chromatid as a template (reviewed in [80]). However, the lack of need for a DNA template in NHEJ does increase the likelihood of nucleotide insertions, deletions and translocation, driving genomic instability [81]. For instance, Lamin A/C depleted U-2OS cells show impaired ability to undergo APE1-mediated DNA incision and POLβ-mediated DNA polymerisation during base excision repair of oxidised DNA [82]. Furthermore, Lamin A/C has been shown to have a role in DSB repair in mouse models. Specifically, knockout of Lamin A/C significantly decreased 53BP1 expression at 1-h post irradiation, with this being predominately due to the upregulation of nuclear and lysosomal Cathepsin L activity [83]. This increased Cathepsin L activity results in the degradation of 53BP1, in addition to pRb and p107. Given 53BP1 has a key role in the facilitation of non-homologous end joining (NHEJ), Lamin A/C-deficient cells are unable to effectively complete NHEJ. In addition, the collective decrease in pRb and p107 expression allows for the binding of the p130/E2FA protein complex, which directly inhibits BRCA1 and RAD51 function. Consequently, the simultaneous BRCA1 and RAD51 downregulation inhibits homologous recombination repair [83]. Based on these findings, it is possible that targeting Lamin A/C overexpression in certain tumour subtypes may impair the cellular DNA damage response; therefore, conferring sensitivity to anti-cancer therapies which induce DNA damage.

In addition, findings from our team have identified a key role for Banf1 in the repair of DNA damage induced by oxidative stress. Initially, it was observed that Banf1 relocalised from the nuclear envelope to the nucleus following treatment with the oxidative stress inducing agents, H_2_O_2_ and Potassium Bromate. Subsequently, it was shown that Banf1 directly bound to PARP1 following oxidative stress to inhibit the auto-ADP-ribosylation of PARP1 and PARP1’s histone ADP-ribosylation, thereby impairing the repair of oxidative lesions [77]. In addition, it has also been observed that siRNA-mediated depletion of Banf1 significantly decreases HR activity and upregulates NHEJ due to Banf1’s role in the regulation of DNA-PK activity [78].

More recently, a novel role for the Lem domain protein, Lemd2, in nucleotide excision repair has been revealed. Nucleotide excision repair is the most frequently utilised pathway to repair bulky, helix distorting DNA lesions induced by ultraviolet light and other environmental mutagens. Although the underlying mechanism remains to be understood, it has been shown that siRNA-mediated depletion of Lemd2 enhances the anti-proliferative effect of ultraviolet-C irradiation. Lemd2-depleted cells also displayed increased γ-H2AX foci at 48 h after UV-C exposure suggesting that Lemd2 may have a role in promoting repair of these lesions [79].

The nuclear pore complexes (NPC), spanning the nuclear periphery, have also been implicated to have a role in the repair of a subset of DNA double-strand breaks [84]. Initially, these protein complexes were shown to induce sensitivity to DNA damaging agents in yeast deletion mutants, suggesting a potential role in DNA repair [64, 65]. Subsequently, it was shown that a subset of HO-induced double-strand breaks moved to the edge of the nucleus and were localised with NPCs on the nuclear periphery [22, 85]. This relocalisation of DNA double-strand breaks was later found to be dependent upon the nucleoporins NUP107, NUP153 and NUP160 in Drosophila cells [86]. While the relocalisation of double-strand breaks has been observed in several cell systems, it should be noted that only a small number of these breaks are relocalised, so it remains to be elucidated whether targeting these proteins would have a substantial effect on DNA repair in tumour cells.

In light of the evidence discussed above, when considering the potential for cancer therapies that target the nuclear envelope it is worth considering that due to the roles of some nuclear envelope proteins in DNA repair, these therapies may prove effective in combination with current DNA repair focused therapeutics, such as PARP inhibitors [87].

In summary, this demonstrates that the nuclear envelope is involved in extensive list of cellular processes, the majority of which are frequently dysregulated in cancer cells. Therefore, nuclear envelope proteins may provide a novel class of targeted anti-cancer therapies by selectively disrupting the nuclear envelope in tumour cells.

## Targeting of nuclear envelope proteins in cancer

Due to the frequently abnormal structure of cancer cell nuclei, it has been suggested that targeting the post-mitotic nuclear assembly and nuclear organisation represent attractive targets for next-generation anti-cancer therapies [36]. Despite this, to date, anti-cancer therapies that specifically target the nuclear envelope proteins have not been developed. However, given the diverse functions of the nuclear envelope proteins in tumour development, it is evident that a nuclear envelope targeted cancer therapy may improve patient outcomes.

Interestingly, the involvement of several nuclear envelope proteins in premature ageing syndromes may offer insight into the capacity of these proteins to be targeted as anti-cancer therapies. For instance, it has been shown that the Lamin A mutation which results in the premature ageing disease Hutchinson-Gilford progeria syndrome impairs cell cycle progression in fibroblasts, most predominately due to impaired Rb hyperphosphorylation and subsequent entry into the S phase [88]. Notably, this is a p53 independent mechanism of inducing cell cycle arrest, which is one of the most frequently mutated genes in tumour cells [89]. Collectively, these findings suggest that the silencing of Lamin A activity may inhibit tumourigenesis in p53-proficient cells by inhibiting progression through the cell cycle. Similarly, several progeria syndromes associated with mutations in nuclear envelope proteins have been linked to decreased activity of several DNA repair pathways, including HR, NER and NHEJ [77, 90]. Targeting the DNA repair pathways has shown promising outcomes as an anti-cancer therapy, particularly in instances of synthetic lethality. Given several DNA repair mechanisms are deficient in several NE-associated premature ageing syndromes, it can be suggested that silencing of these NE proteins may provide effective monotherapies or in combination with other DNA repair targeted cancer therapies.

In addition, although there is yet to be the development of an anti-cancer therapy which targets the nuclear envelope proteins, drugs targeting nucleocytoplasmic export and import have shown promising outcomes in clinical and preclinical trials human and veterinary trials [91]. For instance, Leptomycin B is an anti-fungal agent known to inhibit nuclear export and has been shown to have anti-cancer activity in lung, cervical and gastric cancer cell models [92]. However, Leptomycin B and its subsequent derivatives were shown to have substantial off-target toxicity and adverse side effects due to the lack of tumour specificity through targeting the NPCs. Following this, several semi-synthetic and synthetic compounds were developed in attempt to target the NPCs, including CBS9106, KOS-2464 and several selective inhibitors of nuclear export (SINES). Studies investigating the anti-cancer activity of these compounds produced conflicting results, demonstrating that targeting the NPCs had strong anti-cancer activity; however, also had considerable off-target toxicity [93–95]. Despite the significant off-target toxicity induced by NPC targeted cancer therapeutics, the anti-cancer activity of targeting the NPCs highlights the importance of a structurally sound nuclear envelope for tumourigenesis. It is suggested that targeting the INM may have less off-target toxicity than targeting the NPCs as the proteins of the INM are more differentially expressed in tumour cells in comparison to non-cancerous cells. For instance, disrupting the NPCs or depleting Lamin A/C expression has been shown to have comparable growth inhibiting effects on non-small cell lung cancer cells undergoing malignant transformation [96]. However, unlike the NPCs, it has been demonstrated that Lamin A/C expression is elevated in several subtypes of cancer [49].

Given tumour cells often demonstrate an aberrant nuclear morphology, it can be hypothesised that the development of an anti-cancer therapy which impairs nuclear envelope integrity in tumour cells may have profound effects. Furthermore, as highlighted within this review, the nuclear envelope proteins are extensively involved in the hallmarks of cancer, suggesting a nuclear envelope targeting cancer drug has the potential to have a potent, multifaceted mechanism of inducing cell death in tumour cells.

## Conclusion

In summary, whilst a nuclear envelope targeting cancer therapy remains to be developed, there is significant evidence to suggest such a drug may have potent anti-cancer activities. Furthermore, it is evident that the nuclear envelope has an essential role in tumourigenesis and, advancing our knowledge of the nuclear envelope proteins and their cellular functions will significantly improve our understanding of tumourigenesis.
